# Radio Signal Recognition Using Two-Stage Spatiotemporal Network with Bispectral Analysis

**DOI:** 10.3390/s25175449

**Published:** 2025-09-03

**Authors:** Hongmei Bai, Siming Li, Yong Jia, Bowen Xiao

**Affiliations:** 1 College of Mechanical and Electrical Engineering, Chengdu University of Technology, Chengdu 610059, China; 202201130113@cudt.edu.cn (H.B.); jiayong2014@cdut.edu.cn (Y.J.); 202320030123@cudt.edu.cn (B.X.); 2 School of Computer and Cyber Security, Chengdu University of Technology, Chengdu 610059, China

**Keywords:** Radio Frequency Signal Recognition, bispectral analysis, spatiotemporal feature extraction, deep learning, LSTM network

## Abstract

With the rapid proliferation of unmanned aerial vehicles (UAVs), reliable identification based on radio frequency (RF) signals has become increasingly important for both civilian and security applications. This paper proposes a spatiotemporal feature extraction and classification framework based on bispectral analysis. Specifically, bispectral estimation is used to convert one-dimensional RF signals into two-dimensional bispectrum feature maps that capture higher-order spectral characteristics and nonlinear dependencies. Based on these characteristics, a two-stage network was constructed for spatiotemporal feature extraction and classification. The first stage utilizes a ResNet18 network to extract spatial structural features from individual bispectrum maps. The second stage employs an LSTM network to learn temporal dependencies across the sequence of bispectrum maps, capturing the continuity and evolution of signal characteristics over time. The experimental results on a public dataset of UAV RF signals show that this method improves recognition accuracy by 6.78% to 13.89% compared to other existing methods across five categories of UAVs.

## 1. Introduction

Unmanned aerial vehicle (UAV) technology has experienced rapid growth recently, finding broad applications in various domains, including agricultural irrigation, aerial mapping, emergency rescue, and power line inspection [1,2,3]. However, with the proliferation of civilian and commercial UAVs, new challenges such as unauthorized incursions by malicious UAVs, airspace violations, and increasing difficulty in real-time monitoring pose serious threats to public safety and airspace security [4].

Reliable UAV identification and tracking technologies have become increasingly vital to mitigating these risks. While traditional approaches based on optical imaging or radar have achieved some success, they often struggle in complex electromagnetic environments or under adverse conditions such as low visibility and heavy clutter. In contrast, radio frequency (RF) signal-based identification has emerged as a promising alternative due to its passive nature, long-range coverage, and resilience to environmental interference [5,6]. As the primary carriers for UAV communication, control, and navigation, RF signals offer unique advantages, including high reliability, non-line-of-sight detection, and robustness to weather variations. These properties make RF-based sensing particularly suitable for UAV identification in complex environments [7,8,9]. Unlike optical and radar systems, RF analysis allows for monitoring without actively sending signals, making it useful for both civilian and military purposes over long distances [10,11]. Therefore, extracting effective RF features and building reliable classifiers is key to enhancing UAV recognition and ensuring safe airspace management in tasks such as drone defense and traffic monitoring [5,9].

Currently, RF-based UAV identification methods can be broadly categorized into three groups: time-domain, frequency-domain, and time–frequency domain approaches, each with its own advantages and limitations. Time-domain methods extract features directly from the waveform, utilizing techniques such as waveform shape analysis and autocorrelation. These approaches are computationally efficient and highly sensitive to burst signals, making them suitable for scenarios with limited hardware resources or real-time constraints. However, their performance tends to degrade significantly under low signal-to-noise ratio (SNR) conditions, and they are highly susceptible to interference, limiting their robustness in complex environments [12]. Frequency-domain methods analyze the statistical distribution of spectral components. Some studies focus on frequency-hopping signals by capturing variations in spectrum spacing and power distribution, while others identify periodic spectral patterns associated with image transmission signals [9]. For example, Lee et al. [13] transformed the recognition of frequency-hopping signals into an image classification task, achieving promising results. However, these signals are inherently sensitive to noise, and the accuracy of such methods may deteriorate rapidly in congested or dynamic spectral environments. Although frequency-domain methods generally outperform time-domain techniques in terms of robustness and accuracy, they still struggle with generalization and noise resilience. Time–frequency domain methods attempt to combine the strengths of both temporal and spectral analyses, enabling a more comprehensive characterization of RF signals. By capturing how frequency content evolves, these approaches offer superior recognition performance, particularly in noisy or time-varying conditions. For instance, the method proposed in [14] converts RF signals into time–frequency spectrograms and applies visual statistical feature matching using sliding windows to recognize UAV types. Although the method is interpretable and lightweight, its dependence on manual feature design and repeated window searches leads to high computation, limiting real-time use.

To address these challenges, recent advances have focused on deep learning-based approaches that automatically learn high-level representations from spectrograms to distinguish RF signals. Multi-scale convolutional neural networks (CNNs), residual networks (ResNet), and other architectures have been employed to extract discriminative features across temporal and spectral scales. For example, Mandal et al. [15] proposed a multi-resolution CNN model that effectively enhances sensitivity and recognition accuracy; Zhao et al. [16] adopted ResNet for classifying two-dimensional time–frequency images of RF signals, achieving precise UAV identification; and Nguyen et al. [17] developed the WRIST system based on a deep learning framework, attaining approximately 90% identification accuracy, which validates the feasibility of such models in practical applications.

Despite performance gains, deep learning models can still pick up noise or artifacts in low-SNR or limited-data settings, which weakens their interpretability and stability [18,19]. To address this, recent research has turned to hybrid architectures combining CNNs with recurrent structures like LSTM. This approach has proven effective in domains relying on spatiotemporal dependencies, such as acoustic event detection [20], human activity recognition [21], and EEG-based emotion classification [22]. In these architectures, CNNs serve as powerful feature encoders that reduce dimensionality and extract local invariant patterns, while LSTMs specialize in learning long-term temporal relationships from sequential inputs. Such hybrid models have also demonstrated potential in RF signal processing tasks, enabling systems to simultaneously capture instantaneous signal characteristics and time-evolving patterns [23].

Inspired by these developments, this paper proposes a spatiotemporal RF recognition framework that combines bispectral analysis and ResNet18-LSTM to capture high-order features and temporal patterns. The proposed method brings spatiotemporal pattern recognition and multimodal feature fusion into RF identification, drawing on their successful use in radar-RF fusion [24], UAV surveillance [25], and wearable sensor analysis [26]. The core innovation lies in introducing bispectral representations as a new modality for deep sequence modeling, enabling the network to jointly learn structural features and temporal dependencies in UAV RF signals.

As a third-order spectral analysis tool, the bispectrum captures both amplitude and phase coupling relationships among frequency components, making it particularly suitable for characterizing complex signal structures and higher-order statistical properties [27,28,29]. This contrasts sharply with traditional methods like the short-time Fourier transform (STFT), which only characterizes linear energy distribution through second-order statistics [27]. The STFT is widely used in RF analysis, but it cannot reflect nonlinear features or high-order relationships. These features are crucial in UAV communication, which often suffers from hardware flaws, signal reflections, and interference. Such distortions manifest as intermodulation products, modulation artifacts, and non-Gaussian noise, appearing as localized high-energy clusters or isolated spectral peaks in the bispectrum. These discriminative features remain entirely invisible in STFT representations [30], rendering bispectral analysis particularly effective for RF signal recognition in complex electromagnetic environments [31].

In the proposed method, bispectrum representations are extracted from segmented RF signals and then processed by a hybrid ResNet18-LSTM architecture. The model can effectively capture spatial features from bispectral images and temporal dependencies across signal frames. This architecture enables end-to-end learning of nonlinear and high-order statistical features directly from raw RF data, outperforming traditional handcrafted methods and shallow classifiers. The experimental results on a publicly available dataset demonstrate that the proposed method achieves superior UAV-type recognition performance compared to existing techniques. To support the development and evaluation of the proposed approach, UAV RF signals were collected using the USRP-2955 software-defined radio platform (National Instruments, Austin, TX, USA). The captured signals primarily operate in the 2.4 GHz and 5.8 GHz ISM bands and were recorded from multiple drone models and remote controllers under controlled conditions. Each segment was annotated with the corresponding UAV type, providing a reliable basis for supervised training and evaluation.

This work presents the following key highlights:

- Integration of bispectral theory for enhanced RF signal robustness: By using higher-order spectral (bispectral) analysis, this method successfully identifies nonlinear phase coupling, making it more resistant to noise and complicated electromagnetic situations.
- Two-stage spatiotemporal network design: A hybrid ResNet18-LSTM architecture is developed to jointly model spatial structures using bispectrum maps and temporal dynamics across frames, significantly boosting recognition accuracy.
- Improved feature discrimination and interpretability: t-SNE visualization shows clearer class boundaries and more compact clusters, indicating better separability and model interpretability.

## 2. RF Signal Preprocessing

In general, the classification of UAV radio frequency (RF) signals consists of three main steps: signal preprocessing, feature extraction, and classification. In this work, the raw RF signals are first preprocessed through four stages: endpoint detection, wavelet denoising [32], framing and windowing, and bispectrum estimation. Specifically, the denoised continuous RF signal is segmented into multiple frames using a fixed-length time window. Then, bispectrum estimation is performed on each frame using a sliding window approach to generate corresponding bispectral feature maps. These maps serve as high-order statistical representations for the subsequent recognition model.

### 2.1. Wavelet-Based Denoising

To effectively suppress high-frequency noise interference in radar RF signals and improve the accuracy of subsequent bispectral analysis and feature extraction, this study adopts a wavelet denoising method based on the discrete wavelet transform (DWT).

The core idea of wavelet denoising is to decompose the signal into multiple scales using wavelet transform, eliminate high-frequency noise components through thresholding, and then reconstruct the signal. This process achieves both smoothing and fidelity preservation in the original signal. The mathematical expression for the decomposition of a signal x(n) using wavelet transform is given as follows:

(1) $$x\left(n\right)=\sum_{k}A_{k}y_{k}\left(n\right)$$

Let $A_{k}$ denote the wavelet transform coefficients, and let $y_{k}\left(n\right)$ represent the wavelet basis functions, where *k* indicates the scale and position indices.

**Figure 1:**
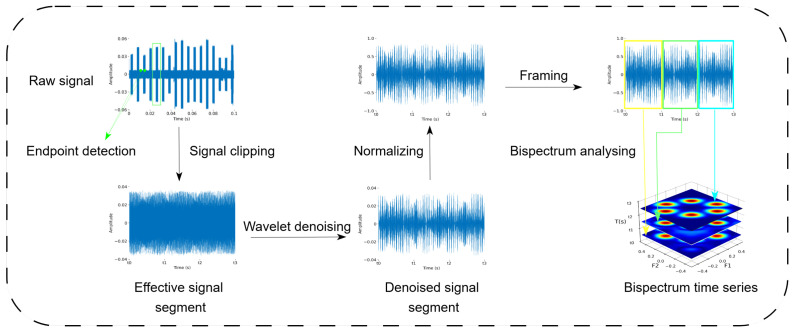
Flowchart of RF signal preprocessing.

The wavelet coefficients $A_{k}$ are computed by projecting the signal x(n) onto the corresponding wavelet basis functions $y_{k}\left(n\right)$. This projection is mathematically expressed as follows:

(2) $$A_{k}=\langle x\left(n\right),y_{k}\left(n\right)\rangle =\int_{-\infty }^{\infty }x\left(n\right)\;y_{k}\left(n\right)\;dn$$

where *k* denotes the scale and position parameters that define the dilation and translation of the wavelet basis functions. Specifically, $y_{k}\left(n\right)$ is derived from a mother wavelet by scaling and shifting to capture localized time–frequency features of the signal, see Figure 1.

After decomposing the signal into wavelet coefficients, a thresholding process is applied to the high-frequency components to suppress noise. To mitigate the discontinuities and distortion often caused by severe thresholding, this study adopts the soft thresholding technique. The soft thresholding function is defined as follows:

(3) $${\hat{d}}_{k}=\mathrm{sgn}\left(d_{k}\right)\cdot \max\left(|d_{k}|-T,0\right)$$

In the above equation, $d_{k}$ denotes the original wavelet coefficient, ${\hat{d}}_{k}$ represents the coefficient after thresholding, *T* is the predefined threshold, and sgnrefers to the sign function.

After thresholding, the denoised signal is reconstructed by applying the inverse discrete wavelet transform (IDWT) on both the low-frequency and thresholded high-frequency coefficients. The reconstruction process can be mathematically expressed as follows:

(4) $$\hat{x}\left(n\right)=\mathrm{IDWT}\left({\hat{A}}_{j},{\hat{D}}_{j}\right)$$

In the above equation, ${\hat{A}}_{j}$ and ${\hat{D}}_{j}$ represent the denoised approximation (low-frequency) and detail (high-frequency) wavelet coefficients, respectively.

In practical implementation, the complex-valued signal is separated into its real and imaginary components, which are independently processed through wavelet decomposition and soft thresholding. The denoised components are then recombined via inverse wavelet reconstruction to obtain a purified complex signal $\hat{x}\left(n\right)$ for subsequent analysis.

After wavelet denoising, the RF signal is segmented into 1024-point temporally continuous blocks, each of which is then processed using bispectral estimation to produce a bispectrum image. These bispectrum images, arranged in time order, form a bispectrum time series that serves as the input to the spatiotemporal neural network.

### 2.2. Framing and Windowing

To satisfy the stationarity assumption required for bispectral estimation and to suppress spectral leakage during the Fourier transform process, framing and windowing are applied to the original signal. The framing operation effectively mitigates boundary effects in signal processing and improves the stability of spectral estimation. Moreover, the selection of an appropriate window function helps enhance spectral characteristics and improves time–frequency resolution.

Assuming the length of the signal to be processed is *L*, with a frame length of *N* and a frameshift of *S*, the total number of frames *M* can be calculated as follows:

(5) $$M=\left\lfloor \frac{L-N}{S}\right\rfloor +1$$

To maximally preserve the original signal information and avoid signal distortion caused by window function weighting, a rectangular window is applied to each frame during the preprocessing stage of bispectral estimation. The rectangular window function is defined as follows:

(6) $$w\left(n\right)=\left\{\begin{array}{cc} 1, & 0\leq n<N\\ 0, & \mathrm{otherwise} \end{array}\right.$$

The rectangular window maintains a constant value within each frame and does not impose any additional weighting on the signal, making it suitable for scenarios where statistical stability is prioritized. In contrast, to reduce spectral leakage and suppress sidelobe interference during the subsequent time–frequency analysis stage, this study adopts the Hamming window, which exhibits favorable sidelobe suppression characteristics. The Hamming window function is defined as follows:

(7) $$w\left(n\right)=0.54-0.46\cos\left(\frac{2\pi n}{N-1}\right)$$

### 2.3. Bispectrum Analysis

To capture nonlinear interactions in UAV RF signals, this study adopts bispectral analysis, a higher-order spectral method that retains amplitude, frequency, and phase information while effectively suppressing Gaussian noise. The following steps detail the bispectrum estimation process adopted in this work.

To adapt to the requirements of the bispectrum-based RF signal recognition task, this study adopts a non-parametric estimation method. Specifically, the denoised signal $\hat{x}\left(n\right)$ is assumed to be a zero-mean normalized sequence sampled at a frequency of $f_{s}$. To enhance the statistical stability of the estimation and satisfy the approximate stationarity assumption, the entire signal is divided into *K* overlapping sub-frames, each containing *M* samples. These sub-frames are denoted as ${\hat{x}}^{\left(k\right)}\left(n\right)$, where k=1,2,…,K and n=0,1,…,M−1. The specific process of frame shifting between adjacent sub-frames is detailed in the previous section on Framing and Windowing.

For each sub-frame, the Discrete Fourier Transform (DFT) is computed as follows:

(8) $$X^{\left(k\right)}\left(\lambda \right)=\frac{1}{M}\sum_{n=0}^{M-1}x^{\left(k\right)}\left(n\right)e^{-j2\pi n\lambda /M}$$

Next, based on the DFT coefficients of each segment, the third-order cumulant is constructed to capture the nonlinear interactions between different frequency components. Its expression is given by:

(9) $${\hat{b}}_{k}\left(\lambda_{1},\lambda_{2}\right)=\frac{1}{\Delta_{0}^{2}}\sum_{i_{1}=-L_{1}}^{L_{1}}\sum_{i_{2}=-L_{1}}^{L_{1}}X^{\left(k\right)}\left(\lambda_{1}+i_{1}\right)\cdot X^{\left(k\right)}\left(\lambda_{2}+i_{2}\right)\cdot X^{\left(k\right)}\left(-\lambda_{1}-\lambda_{2}-i_{1}-i_{2}\right)$$

Here, ${\hat{b}}_{k}\left(\lambda_{1},\lambda_{2}\right)$ represents the third-order Fourier correlation estimate of the *k*-th segment at the frequency pair $\left(\lambda_{1},\lambda_{2}\right)$. $i_{1}$ and $i_{2}$ denote frequency shift indices. $\Delta_{0}=f_{s}/N_{0}$ is the frequency sampling interval. $L_{1}$ and $N_{0}$ satisfy the constraint $M=\left(2L_{1}+1\right)N_{0}$. The frequency indices must satisfy $0\leq \lambda_{2}\leq \lambda_{1}$ and $\lambda_{1}+\lambda_{2}\leq f_{s}/2$.

By averaging the third-order correlation spectra of all segments, the bispectrum estimate of the entire signal is obtained as follows:

(10) $${\hat{B}}_{D}\left(\omega_{1},\omega_{2}\right)=\frac{1}{K}\sum_{k=1}^{K}{\hat{b}}_{k}\left(\omega_{1},\omega_{2}\right)$$

The resulting ${\hat{B}}_{D}\left(\omega_{1},\omega_{2}\right)$ forms the bispectral map on the two-dimensional frequency plane $\left(\omega_{1},\omega_{2}\right)$, where each element represents the third-order phase coupling strength between a pair of frequency components. This reflects the intrinsic nonlinear characteristics of the signal and provides a basis for subsequent high-order feature extraction and recognition modeling.

## 3. Two-Stage Spatiotemporal Network

This study proposes a spatiotemporal framework using bispectrum images that combines CNN and RNN to effectively capture nonlinear higher-order features in RF time-series signals. The framework consists of two main stages:

In the first stage, the study employs a ResNet18 network as the spatial feature extractor, taking into account the spatial structural features of the bispectrum in the two-dimensional frequency domain. Each frame of the bispectrum image is encoded via convolution to thoroughly capture local details and global statistical patterns. This stage outputs a series of spatial feature vectors that form a high-level temporal representation.

**Figure 2:**
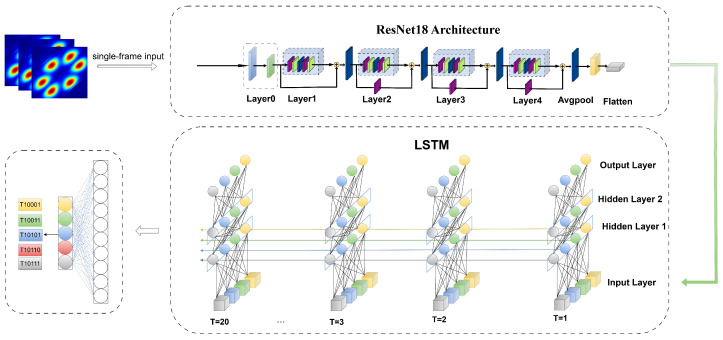
ResNet-LSTM network architecture.

In the second stage, a long short-term memory (LSTM) network is introduced to further model the dynamic evolution of bispectral features across different time segments and to perform temporal modeling on the spatial feature sequence. The LSTM effectively captures inter-frame contextual dependencies and state changes, extracting deep semantic information along the time dimension. Ultimately, the LSTM output temporal features are used for classification, enabling accurate recognition of non-stationary RF signals. The proposed network architecture is illustrated in Figure 2.

### 3.1. Residual Network

**Figure 3:**
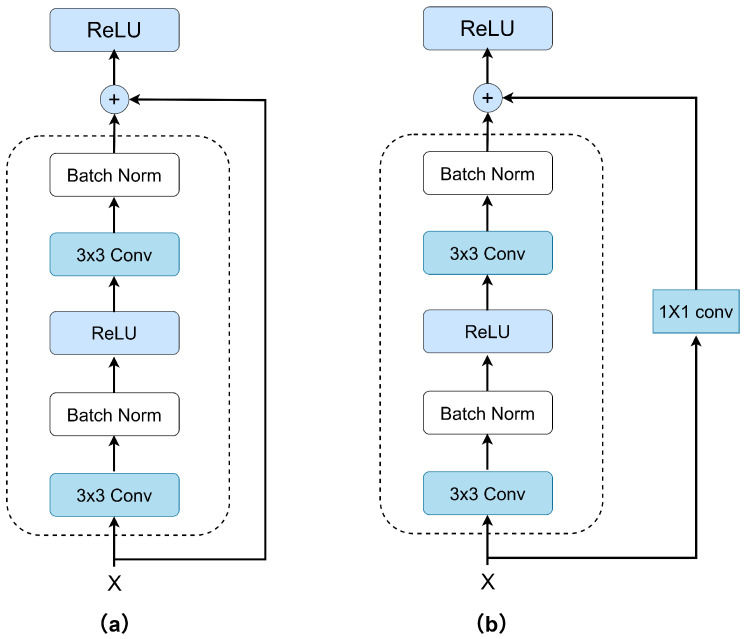
Standard residual block and residual block with downsampling layer [34]. (**a**) Standard residual block. (**b**) Residual block with downsampling layer.

In this study, the ResNet18 network was adopted as the backbone architecture for the spatial feature extraction module. ResNet18 is constructed by stacking multiple residual blocks, and its overall architecture consists of an input layer, multiple convolutional (Conv) layers, batch normalization (BN) layers, nonlinear activation (ReLU) layers, skip connections, fully connected (FC) layers, and an output layer. Conventional deep convolutional neural networks often suffer from gradient vanishing or explosion as the network depth increases. ResNet [33] introduces a residual connection mechanism, where the input feature *x* is added directly to the output of a convolutional block F(x) through an identity mapping. The residual formulation can be expressed as follows:

(11) $$y=F(x,\left\{W_{i}\right\})+x$$

where $F(x,\left\{W_{i}\right\})$ represents the nonlinear transformation function composed of convolutional layers, batch normalization, and ReLU activation within the residual block. *x* is the input, and *x* is the output of the block. This skip connection structure effectively alleviates degradation issues during gradient propagation, making the network easier to train and more capable of learning complex feature representations. Specifically, in ResNet18, each residual block consists of two 3×3 convolutional layers, enabling the extraction of multi-scale spatial features. In this work, ResNet18 is utilized to process the input two-dimensional bispectrum feature maps. Through layer-by-layer convolutional operations, both local details and global spatial structures are extracted, resulting in discriminative spatial feature representations. These spatial features are then fed into the subsequent temporal modeling module to accomplish RF signal recognition. A typical residual block structure of ResNet18 is illustrated in Figure 3.

### 3.2. Long Short-Term Memory Network

**Figure 4:**
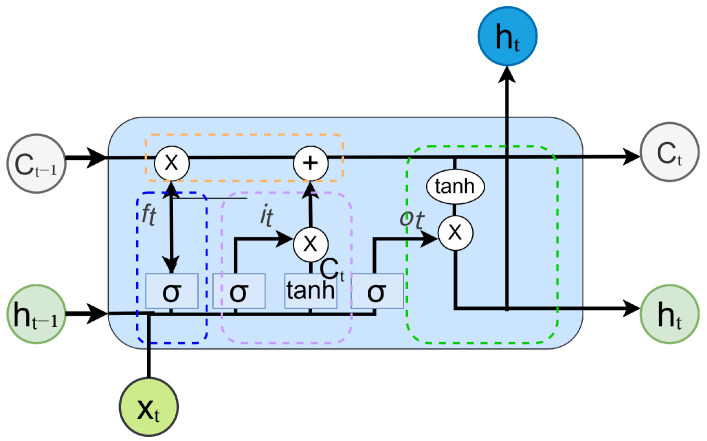
Structure of the LSTM cell.

The long short-term memory (LSTM) network is an improved variant of the recurrent neural network (RNN) specifically designed for handling sequential data. Proposed by Hochreiter et al. in 1997 [35], LSTM has been widely applied to tasks involving sequence modeling and feature extraction. Traditional RNNs suffer from the vanishing or exploding gradient problem during backpropagation, making them less effective in capturing long-term dependencies. To address this limitation, LSTM introduces a gating mechanism comprising a forget gate, input gate, and output gate, which enables selective memory retention and updating of information. This mechanism significantly enhances the model’s ability to capture long-range dependencies, thereby improving its prediction accuracy and feature extraction performance. Given that LSTM is capable of learning long-term dependencies in sequential data, it is well suited for extracting temporal features from RF signals. In this study, the bispectral feature maps of RF signals exhibit sequential characteristics across consecutive time frames, making them compatible with LSTM-based temporal modeling. Figure 4 illustrates the structure of a standard LSTM cell.

The LSTM unit mainly consists of three key gates, namely, the input gate, the forget gate, and the output gate. The structure is explained in detail below.

The input gate controls whether the current input information should be written to the memory cell. It determines which parts of the current input $x_{t}$ and the previous hidden state $h_{t-1}$ should be used to update the current memory. This process is described by Equation (12):

(12) $$i_{t}=\sigma \left(W_{i}\left[h_{t-1},x_{t}\right]+b_{i}\right)$$

The forget gate determines how much of the previous state information should be retained. Specifically, it regulates the proportion of the previous memory cell state $C_{t-1}$ that is preserved in the current update. This mechanism is formulated as Equation (13):

(13) $$f_{t}=\sigma \left(W_{f}\left[h_{t-1},x_{t}\right]+b_{f}\right)$$

The output gate decides how much information from the current cell state should be output at the present step. This operation is described by Equation (14):

(14) $$o_{t}=\sigma \left(W_{o}\left[h_{t-1},x_{t}\right]+b_{o}\right)$$

Within the gating structure, a combination of the sigmoid activation function and the tanh function is commonly employed. The sigmoid function maps the input to a range of [0, 1], allowing it to control the proportion of information that is retained or discarded. In contrast, the tanh function restricts values to the range [−1, 1], which enhances the network’s ability to model nonlinear relationships effectively.

### 3.3. ResNet18-LSTM Network

The proposed ResNet18-LSTM network architecture consists of two cascaded components: ResNet18 and LSTM. The changes in input tensor dimensions throughout the model are summarized in Table 1. The rectified linear unit (ReLU) is adopted as the activation function to introduce non-linearity and improve model expressiveness. For pooling operations, the model uses max pooling at the beginning and average pooling at the end to improve feature extraction and overall robustness.

## 4. Experimental Setup and Results

### 4.1. Dataset Description

The dataset utilized in this study is DroneRFa [14], released in 2024 by the College of Information Science and Electronic Engineering at Zhejiang University. It serves as a standardized benchmark for UAV radio frequency (RF) signal recognition and classification. DroneRFa contains RF recordings from 24 types of commonly used UAVs and flight controllers, including brands such as DJI, VBar, FrSky, and Futaba, along with a background class representing non-UAV activity. All signals were captured using the USRP-2955 platform (National Instruments, Austin, TX, USA), which supports four-channel transceiving with up to 80 MHz bandwidth and a 100 MS/s sampling rate. The signals primarily occupy the 2.4 GHz and 5.8 GHz bands, with each sample lasting 1 to 2 s. The dataset also includes multi-dimensional annotations, such as the UAV model, acquisition distance, and signal segment index, enabling tasks like classification, detection, and behavior analysis.

In this study, five representative UAV signal types were selected from the DroneRFa dataset: VBar (VBar, Hallbergmoos, Germany, T10001), Futaba T6IZ (Futaba, Osaka, Japan, T10011), RadioLink AT9S (RadioLink, Shenzhen, China, T10101), Futaba T14SG (Futaba, Osaka, Japan, T10110), and Yunzhuo T12 (Yunzhuo, Beijing, China, T10111). These devices were intentionally chosen to ensure diversity in both manufacturers and communication protocols. Specifically, the selected flight controllers originate from distinct brands across Germany, Japan, and China, including both high-end and consumer-grade systems. They employ different modulation schemes such as the frequency-hopping spread spectrum (FHSS), direct-sequence spread spectrum (DSSS), Gaussian frequency-shift keying (GFSK), and proprietary digital protocols. All five selected types of UAV RF signals were collected at comparable distances ranging from 20 to 40 m, ensuring consistency across acquisition conditions. The diversity in device type, protocol, and acquisition conditions leads to distinct waveform and spectral characteristics, making these five types representative of the broader signal diversity in the full dataset. Therefore, the selected subset provides a meaningful and practical basis for evaluating the generalization performance of the proposed method.

### 4.2. Experimental Environment and Parameter Settings

The bispectral estimation in this study was conducted using MATLAB R2022b. The core neural network algorithms were implemented in the PyCharm 2024 Community Edition environment, using Python 3.8 as the programming language. All neural network classification models were developed based on the PyTorch framework. The hardware platform used for model training included an Intel i7-10700K CPU, an NVIDIA GeForce RTX 3090 GPU, and 24 GB of RAM. Regarding the model configurations, for the ResNet18-LSTM network, the input image size was set to 224 × 224, with a learning rate of 1 × 10^−4^, a batch size of 4, and a total of 100 training epochs. To prevent overfitting during training, the model employed the Adam optimizer.

### 4.3. Bispectral Feature Map Acquisition

**Figure 5:**
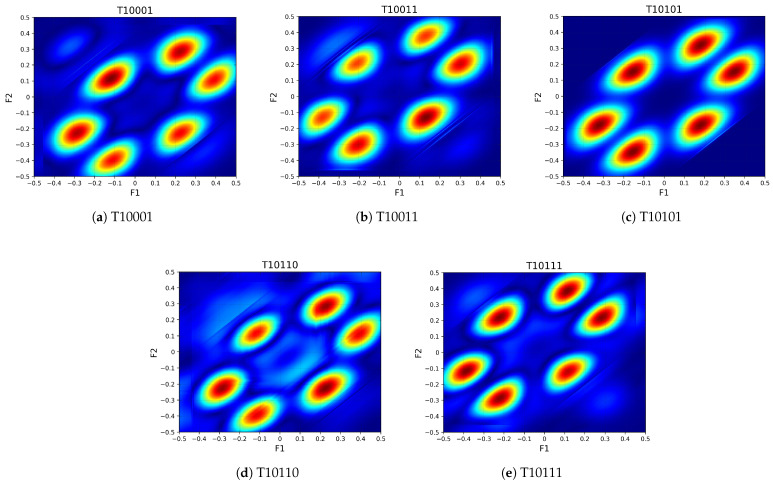
Representative bispectral feature maps of five UAV RF signal categories: (**a**) T10001, (**b**) T10011, (**c**) T10101, (**d**) T10110, (**e**) T10111. F1 and F2 represent normalized frequency components in the bispectrum domain, with values ranging from –0.5 to 0.5.

In this study, bispectral feature maps were constructed based on the RF signals from the DroneRFa dataset. Specifically, five representative types of UAV radio frequency signals (T10001, T10011, T10110, T10101, and T10111) were selected for analysis. Each signal type underwent a series of preprocessing procedures tailored for RF signal analysis. During the framing and windowing stage, the signal length was set to L = 1024, with a frame length of N = 512 and a frame step of M = 10. A Hamming window was applied to each frame to reduce spectral leakage. Subsequently, bispectral estimation was performed on each windowed segment to extract the bispectral representation in the two-dimensional frequency domain. Figure 5 presents typical bispectral patterns corresponding to the five UAV signal types, revealing the distinct high-order spectral characteristics across different categories.

Following this procedure, a large number of temporally continuous bispectral feature maps were generated for each signal class, with each map representing the third-order spectral structure of a signal segment. Every 20 consecutive bispectral maps were grouped to form one complete sample, which was used as the input to the subsequent deep neural network model. Ultimately, 360 valid samples were constructed for each UAV category, supporting both model training and performance evaluation.

### 4.4. Comparison with Other Methods

**Figure 6:**
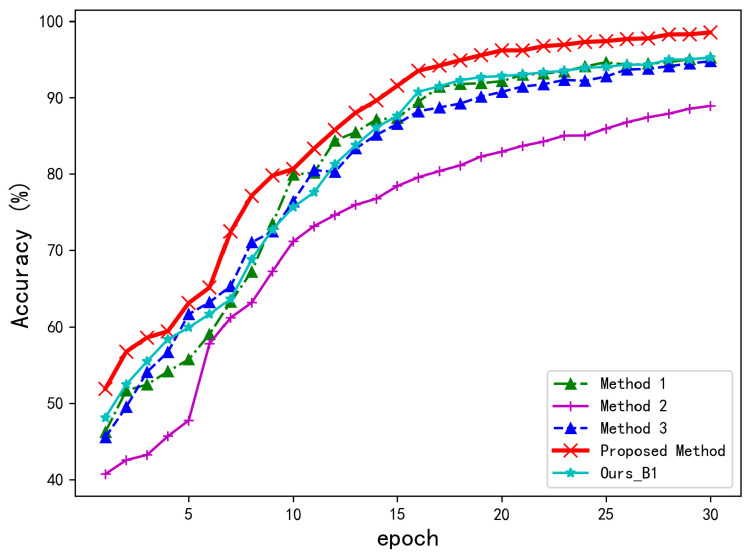
Comparison of recognition accuracy across different methods on the same dataset. Method 1–3 correspond to [37], [36], and [14], respectively.

**Figure 7:**
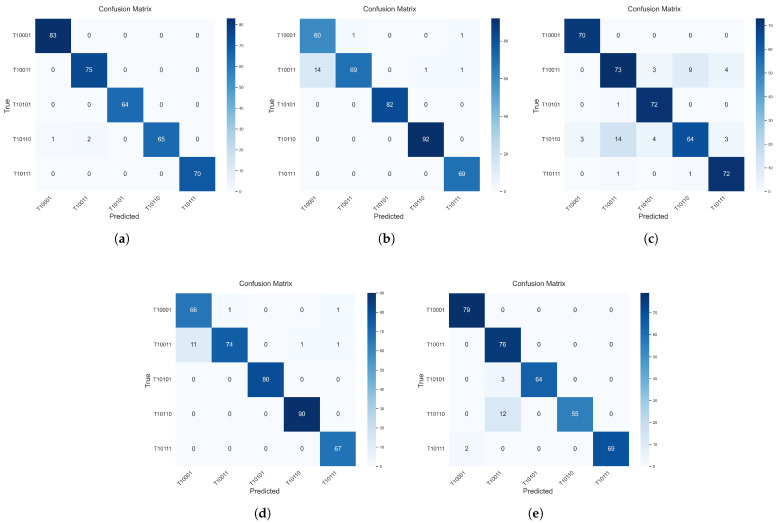
Confusion matrices for five different RF signal identification methods. (**a**) The proposed method is based on bispectral representations and ResNet18-LSTM. (**b**) Method from Ref [14] utilizing STFT features and ResNet18. (**c**) Method from Ref [36] using a single bispectrum frame with an attention module. (**d**) The feature fusion method from Ref [37] combining STFT and bispectrum inputs for ResNet classification. (**e**) Baseline method Ours_B1 using 20 consecutive bispectral frames as input to a ResNet18-LSTM model.

To comprehensively evaluate the performance of the proposed bispectrum-based ResNet18-LSTM model for UAV radio frequency (RF) signal recognition, four comparative experiments with same dataset and training configurations were conducted. These included reproductions of three representative existing methods and one baseline experiment using continuous bispectrum images as input to the ResNet18-LSTM model. The reproduced methods are as follows: a non-sequential classification approach based on single-frame bispectrum images enhanced by an attention module (AM) [36], a time–frequency feature modeling method that generates spectrograms using the short-time Fourier transform (STFT) and performs classification with a ResNet18 network [14], and a multi-feature fusion model that simultaneously integrates STFT spectrograms and bispectrum images into a ResNet-based framework [37]. The baseline experiment utilizes the proposed ResNet18-LSTM architecture with bispectrum images as input, aiming to evaluate the specific contribution of bispectrum features to the proposed method. All models were trained and tested with identical data splits, training epochs, and optimizer settings to ensure the fairness and consistency of the evaluation. The recognition accuracies of each model are presented in Figure 6, and the corresponding confusion matrices are shown in Figure 7.

As illustrated in Figure 6, the proposed two-stage bispectrum-based model achieves a recognition accuracy exceeding 98% with identical dataset and training configurations, demonstrating significant superiority over the four comparative methods. The baseline method we reproduced (Ours_B1), which uses 20 consecutive bispectrograms for each input, reaches about 95% accuracy. Among existing methods, the multi-feature fusion model [37] and STFT-based ResNet18 [14] achieve competitive final accuracies of 94%. Conversely, the attention-enhanced non-sequential model [36] demonstrates consistently inferior performance, plateauing below 90% accuracy.

This performance reveals two critical findings: First, the proposed dual-stage architecture outperforms the identically formulated baseline (Ours_B1) by 3 percentage points, validating the advantage of using consecutive bispectrograms as input. Second, a comparison between the baseline method Ours_B1 and the attention-enhanced non-sequential model [36] confirms the importance of incorporating LSTM-based sequential modeling. The performance gap is particularly pronounced during early training phases, where our method exceeds 90% accuracy within merely 10 epochs, achieving a convergence speed approximately 30% faster than STFT-based counterparts. In contrast, the proposed method benefits from the LSTM module’s ability to capture temporal dynamics and the bispectrum’s capability to encode high-order statistical information. This combination effectively enhances the model’s feature representation and discriminative power, leading to superior learning efficiency and recognition accuracy. To further demonstrate the performance advantage, Table 2 reports the average recognition accuracies achieved by all methods after 5, 10, 15, 20, 25, and 30 training epochs under identical training conditions.

As shown in Table 2, the proposed method achieves an average recognition accuracy of 90.23%, improving by 6.78% over the STFT and bispectrum fusion method [37] and by 7.98% compared to the STFT-based ResNet18 method [14]. Notably, the proposed method achieves a substantial improvement of 13.89% compared to the non-sequential modeling approach that uses a single-frame bispectrum image combined with an attention module [36].

In terms of model complexity, the proposed approach has 12.49 million parameters, which is significantly fewer than the 21.29 million of the STFT-bispectrum fusion method [37], yet it achieves better accuracy. Compared with the baseline variant Ours_B1, the proposed method improves recognition performance by 8.04%, with only a moderate increase in processing time (from 15.73 ms to 23.11 ms per frame) and memory usage. This additional computational cost mainly arises from the bispectral analysis preprocessing, which extracts higher-order nonlinear features essential for enhancing model performance. Considering real-time applicability, the average processing time of approximately 23 ms per frame corresponds to a processing rate of about 43 frames per second, which is generally adequate for many practical RF signal recognition applications with moderate frame rates. Therefore, the trade-off between increased preprocessing time and improved accuracy is reasonable and favorable for real-time or near-real-time deployment, demonstrating the effectiveness of our temporal modeling strategy and bispectral feature representation in RF signal identification tasks.

To further analyze the classification effectiveness of each method across individual UAV categories, Table 3 presents a detailed comparison in terms of precision, recall, and specificity. As shown in the table, the proposed ResNet18-LSTM model achieves perfect precision (100%) across all five UAV classes. Recall is also perfect (100%) for four of the five categories, with a slightly reduced value (95.59%) observed for class T10110. The baseline experiment (Ours_B1) maintains high performance overall, though it shows comparatively lower recall (82.09%) for class T10110 and (95.52%) for T10101. In contrast, Ref [36] exhibits significant instability, with both precision and recall substantially lower than those of other methods in multiple categories, especially T10011 and T10110, which lack sufficient temporal modeling. Meanwhile, Refs [14,37] exhibit moderate performance but still fall short in achieving consistent accuracy across all classes. These results collectively demonstrate the advantage of integrating bispectral representations with LSTM-based temporal modeling, which effectively captures the nonlinear and time-evolving features of RF signals. Furthermore, the consistently high specificity across categories confirms the robustness and discriminative power of the proposed method.

To statistically validate the performance improvements achieved by the proposed ResNet18-LSTM model, Table 4 presents the results of paired t-tests conducted between the proposed method and each of the comparative approaches across four key metrics: accuracy, precision, recall, and specificity. The results consistently show statistically significant differences (all *p*-values < 0.05) in favor of the proposed method. Notably, the largest improvements are observed in comparisons with Ref [36], with particularly high t-values for accuracy (t(4) = 95.49), precision (t(4) = 31.32), and recall (t(4) = 71.31), indicating substantial performance gains. These results align with prior observations that this baseline suffers from inadequate temporal modeling. Compared to Ours_B1, the proposed method also demonstrates significant improvements, especially in recall (t(4) = 37.46) and accuracy (t(4) = 39.05), confirming the benefit of integrating bispectral representations with temporal modeling. In terms of specificity, although the mean differences are smaller, the statistical tests still indicate meaningful gains. Overall, these statistical findings further reinforce the effectiveness and robustness of the proposed model.

### 4.5. Visual Analysis of Learned Features via t-SNE

**Figure 8:**
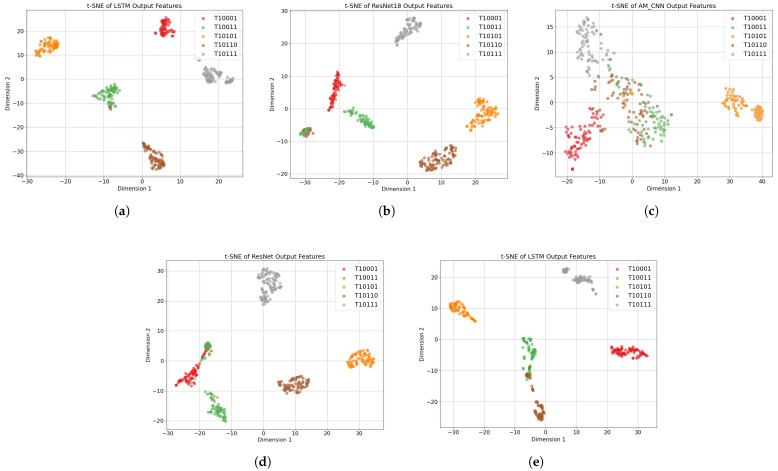
t-SNE visualization of features from five UAV RF signal categories with four different methods. (**a**) The proposed method based on bispectral representations and ResNet18-LSTM. (**b**) Method from Ref [14] utilizing STFT features and ResNet18. (**c**) Method from Ref [36] using a single bispectrum frame with an attention module. (**d**) The feature fusion method from Ref [37] combining STFT and bispectrum inputs for ResNet classification. (**e**) Baseline method Ours_B1 using 20 consecutive bispectral frames as input to a ResNet18-LSTM model.

To qualitatively assess the discriminative power of learned feature representations, this study employs t-distributed stochastic neighbor embedding (t-SNE) [38,39,40] to visualize the output features from five RF signal identification models. Specifically, the feature vectors extracted from each input sample by these models are projected into a two-dimensional space. Different UAV signal categories are denoted using distinct colors [41,42] to reveal the distribution patterns in the learned feature space. The resulting t-SNE scatter plots are presented in Figure 8.

Among these models, the proposed ResNet18-LSTM achieves the most compact and well-separated clustering of UAV categories, with clear class boundaries and minimal overlaps. This indicates that the model effectively captures high-order statistical characteristics and spatiotemporal dependencies from bispectral inputs, leading to highly discriminative representations.

In contrast, the ResNet18 model based on STFT features [14] produces less compact clusters, with noticeable overlaps between categories such as T10001 and T10011. This suggests that while frequency-domain features aid in basic category separation, the lack of temporal modeling limits the model’s capacity to identify subtle inter-class variations. The AM method shown in Figure 8c [36], which leverages single-frame bispectrum representations and an attention module, exhibits the weakest clustering performance. The feature points are widely scattered with significant class overlaps, highlighting the limitations of relying solely on static bispectral frames without temporal or multi-scale context modeling. The multi-feature fusion method illustrated in Figure 8d [37], which integrates both STFT and bispectral features within a ResNet backbone, shows a moderate improvement in cluster separability compared to Figure 8b,c. However, the clusters remain loosely packed, and some category boundaries are still ambiguous. This implies that, although feature fusion enhances representational diversity, the absence of temporal modeling constrains the full exploitation of temporal patterns. Figure 8e shows that the baseline method, which uses continuous time–frequency spectrograms with a ResNet-LSTM, has weaker clustering between categories like T10011 and T10110 compared to our method. This highlights the inherent limitations of time–frequency representations in capturing discriminative temporal features.

Overall, the comparative t-SNE visualizations underscore the superiority of the proposed ResNet18-LSTM model in learning compact, well-separated, and highly discriminative embeddings. This visual evidence further validates the model’s strong classification performance and the effectiveness of integrating bispectral representations with temporal modeling.

## 5. Conclusions

In this paper, a novel UAV radio frequency (RF) signal recognition method is proposed, which integrates bispectral analysis with a deep neural network. By applying bispectral transformation to the RF signals, nonlinear high-order statistical features are effectively extracted. To fully exploit both the spatial structure of bispectrum images and their temporal dependencies, a hybrid ResNet18-LSTM architecture was constructed. This combined network enables accurate classification of multiple types of UAV RF signals. The experimental results demonstrate that the proposed method outperforms existing approaches under the same dataset and training conditions, showing superior recognition performance and promising potential for real-world engineering applications. Future work will extend the evaluation to a larger subset of the DroneRFa dataset, encompassing more UAV types and communication protocols, to further validate the generalizability of the proposed method.

## Figures and Tables

**Table 1:** Network model and feature vector dimensionality changes.

Network Structure	Kernel Size	Output Dimensions	Stride
Input Layer	-	4 × 20 × 3 × 224 × 224	-
Conv Layer 1	7 × 7	80 × 64 × 112 × 112	2 × 2
Maxpool Layer 1	3 × 3	80 × 64 × 56 × 56	2 × 2
Residual Block 1	3 × 3	80 × 64 × 56 × 56	1 × 1
Residual Block 2	3 × 3	80 × 128 × 28 × 28	2 × 2
Residual Block 3	3 × 3	80 × 256 × 14 × 14	2 × 2
Residual Block 4	3 × 3	80 × 512 × 7 × 7	2 × 2
Average Pool 1	1 × 1	80 × 512 × 1 × 1	-
Flatten Layer	-	80 × 512	-
Reshape	-	4 × 20 × 512	-
Permute	-	20 × 4 × 512	-
LSTM Input	-	20 × 4 × 512	-
LSTM Output	-	20 × 4 × 256	-
LSTM[-1]	-	4 × 256	-
Linear	-	4 × 5	-

**Table 2:** Comparison of recognition performance across different methods.

Method	Accuracy (%)	Model Parameters	Average Processing Time per Frame (ms)	Memory Usage (MB)	Source
Method 1	83.45	21.29 M	51.35	213.67	Ref [37]
Method 2	76.34	0.38 M	3.97	51.43	Ref [36]
Method 3	82.25	11.18 M	29.55	100.21	Ref [14]
Ours_B1	82.19	12.49 M	15.73	604.32	Ours_B1
Proposed	90.23	12.49 M	23.11	634.73	This Paper

Note: Memory usage was measured during the inference phase for evaluation mode (model.eval()), reflecting the runtime memory requirement per sample.

**Table 3:** Classification performance comparison of five methods.

Method	Class	Precision (%)	Recall (%)	Specificity (%)
Proposed Method	T10001	98.81	100.00	99.64
	T10011	97.40	100.00	99.30
	T10101	100.00	100.00	100.00
	T10110	100.00	95.59	100.00
	T10111	100.00	100.00	100.00
Ref [14]	T10001	81.08	96.77	95.73
	T10011	98.57	81.18	99.67
	T10101	100.00	100.00	100.00
	T10110	98.92	100.00	99.66
	T10111	97.18	100.00	99.38
Ref [36]	T10001	95.89	100.00	99.07
	T10011	82.02	82.02	94.75
	T10101	91.14	98.63	97.82
	T10110	86.49	72.73	96.73
	T10111	91.14	97.30	97.81
Ref [37]	T10001	85.71	97.06	96.60
	T10011	98.67	85.06	99.67
	T10101	100.00	100.00	100.00
	T10110	98.90	100.00	99.67
	T10111	97.10	100.00	99.38
Ours_B1	T10001	97.53	100.00	99.29
	T10011	83.52	100.00	94.72
	T10101	100.00	95.52	100.00
	T10110	100.00	82.09	100.00
	T10111	100.00	97.18	100.00

**Table 4:** Paired t-test results for performance comparison between the proposed method and other approaches.

Performance	Comparison Groups	*p*-Value	t(4)	Mean Difference (%)
Accuracy	Proposed vs. Ref [14]	<0.05	17.65	3.71
	Proposed vs. Ref [36]	<0.05	95.49	9.90
	Proposed vs. Ref [37]	<0.05	17.38	2.79
	Proposed vs. Ours_B1	<0.05	39.05	3.70
Precision	Proposed vs. Ref [14]	<0.05	14.67	4.35
	Proposed vs. Ref [36]	<0.05	31.32	10.73
	Proposed vs. Ref [37]	<0.05	10.04	4.19
	Proposed vs. Ours_B1	<0.05	9.53	3.34
Recall	Proposed vs. Ref [14]	<0.05	29.66	3.28
	Proposed vs. Ref [36]	<0.05	71.31	9.01
	Proposed vs. Ref [37]	<0.05	16.65	2.60
	Proposed vs. Ours_B1	<0.05	37.46	4.22
Specificity	Proposed vs. Ref [14]	<0.05	9.23	0.70
	Proposed vs. Ref [36]	<0.05	43.24	2.52
	Proposed vs. Ref [37]	<0.05	3.63	0.17
	Proposed vs. Ours_B1	<0.05	6.87	0.67

## Data Availability

The data supporting the findings of this study are derived from the DroneRFa dataset, a large-scale radio-frequency (RF) signal dataset of drones. In this work, we utilized the RF signal recordings of five drone categories from the DroneRFa dataset. This dataset is available from the corresponding author upon reasonable request or can be accessed following the dataset’s official release policies as detailed in Ningning et al. (2024), “DroneRFa: A large-scale dataset of drone radio frequency signals for detecting low-altitude drones,” *J. Electron. Inf. Technol.*, 46(4), DOI: https://jeit.ac.cn/cn/article/doi/10.11999/JEIT230570. Users should follow the dataset’s license and citation requirements when using these data.
